# Efficacy of pharmacotherapies for short-term smoking abstinance: A systematic review and meta-analysis

**DOI:** 10.1186/1477-7517-6-25

**Published:** 2009-09-18

**Authors:** Edward J Mills, Ping Wu, Dean Spurden, Jon O Ebbert, Kumanan Wilson

**Affiliations:** 1 Faculty of Health Sciences, Simon Fraser University, Burnaby, Canada; 2 Department of Epidemiology, LSHTM, UK; 3 Pfizer Limited, Tadworth, UK; 4 Mayo Clinic College of Medicine, Rochester, USA; 5 Department of Medicine, Ottawa Hospital Research Institute, Ottawa, Canada

## Abstract

**Background:**

Smoking cessation has important immediate health benefits. The comparative short-term effectiveness of smoking cessation interventions is not well known. We aimed to determine the relative effectiveness of nicotine replacement therapy (NRT), bupropion and varenicline at 4 weeks post-target quit date.

**Methods:**

We searched 10 electronic medical databases (inception to October 2008). We selected randomized clinical trials [RCTs] evaluating interventions for our primary outcome of abstinence from smoking at at-least 4 weeks post-target quit date, with biochemical confirmation. We conducted random-effects odds ratio (OR) meta-analysis and meta-regression. We compared treatment effects across interventions using head-to-head trials and calculated indirect comparisons.

**Results:**

We combined a total of 101 trials evaluating delivery of NRT versus inert controls at approximately 4 weeks post-target quit date (total n = 31,321). The pooled overall OR is OR 2.05 (95% Confidence Interval [CI], 1.89-2.23, P =< 0.0001). We pooled data from 31 bupropion trials contributing a total n of 11,118 participants and found a pooled OR of 2.25 (95% CI, 1.94-2.62, P =< 0.0001). We evaluated 9 varenicline trials compared to placebo. Our pooled estimate for cessation at 4 weeks post-target quit date found a pooled OR of 3.16 (95% CI, 2.55-3.91, P =< 0.0001). Two trials evaluated head to head comparisons of varenicline and bupropion and found a pooled estimate of OR 1.86 (95% CI, 1.49-2.33, P =< 0.0001 at 4 weeks post-target quit date. Indirect comparisons were: NRT and bupropion, OR, 1.09, 95% CI, 0.93-1.31, P = 0.28; varenicline and NRT, OR 1.56, 95% CI, 1.23-1.96, P = 0.0002; and, varenicline and bupropion, OR 1.40, 95% CI, 1.08-1.85, P = 0.01.

**Conclusion:**

Pharmacotherapeutic interventions are effective for increasing smoking abstinence rates in the short-term.

## Introduction

Smoking remains the leading cause of preventable death in the world.[1] Smoking cessation is associated with important benefits at the individual and societal levels. Given the prevalence of smoking, considerable efforts have been directed toward developing interventions to assist smokers in quitting. However, smoking cessation interventions have had heterogeneous successes.[2] Smoking cessation is necessary to reduce future morbidity and mortality, however many patients have difficulty discontinuing.

Both psychosocial and pharmaceutical interventions have been evaluated for their success in achieving smoking discontinuation.[3,4] Drug therapies are now licensed in North America and Europe to promote smoking cessation. The most commonly evaluated of these has been nicotine replacement therapy [NRT].[5,6] More recently, attention has focused on the use of anti-depressant therapy and specifically the agent bupropion[7]. A new intervention approved in 2006, varenicline, targets nicotine receptors to reduce craving and pleasure sensations. Recent guidelines and evaluations call for combining therapies to provide optimal patient management.[3,8]

We,.[9] and others,. [10-13] have previously reported on the efficacy of these interventions for longer-term cessation (3-12 months) durations. No systematic review has yet evaluated short-term quit rates from available therapies. Guidelines for smoking cessation programmes consider quitting 4-weeks post-planned quit date as a successful short-term cessation.[14] Short-term smoking abstinence is especially important in patients requiring immediate behaviour changes, such as those with recent cardiovascular events.[15] or undergoing surgery.[16] We conducted a meta-analysis of Randomized Clinical Trials [RCTs] to identify the effectiveness of the various pharmacological interventions in improving abstinence rates at 4-weeks and 6 months.

## Methods

### Eligibility Criteria

Our primary outcome of interest was smoking abstinence at approximately 4 weeks post-target quit date (TQD). Our secondary outcomes were short-term smoking abstinence defined as 6 months after initiating treatment or closest available data to that time point, within one month. We included any RCT of NRT of any delivery method, bupropion or varenicline. We included only RCTs of at least 4 weeks duration with biochemical confirmation of smoking abstinence because of the likelihood of abstinence over-reporting. While methods of assessing smoking abstinence vary from study to study, the most common method is self-report. However, this can have false cessation rates as high as 30%.[17]False reporting is most likely to occur in a trial setting or in assessing smoking status after a medical event. Laboratory tests are often used to verify smoking status, especially in clinical trials. Methods of biological verification include serum and saliva thiocyanate (SCN), expired carbon monoxide (CO), plasma, saliva and urinary cotinine and plasma and urinary nicotine. Each of these have various strengths and weaknesses.[18] Studies had to report smoking abstinence as either sustained abstinence at the time periods or point-prevalence of abstinence. When both outcomes were available, we considered sustained abstinence to be a superior clinical marker of abstinence. We excluded dose ranging studies, non-RCTs, post-hoc analyses, maintenance therapy, and studies that reported outcomes as self-report.

### Study endpoints

Our primary endpoint was the 4-week post-TQD. This is variably reported in studies over years of publications. National committees require data on the 4-week post-TQD and each group of trials of intervention deals with this endpoint differently. Newer studies typically report this as the last 4-weeks of treatment as pharmacotherapy is begun prior to TQD. Where this specific endpoint is reported, we extracted data on 4-week post-TQD. Where not reported, we extracted data on 4 weeks post-intervention. Our secondary endpoint, 6-months post intervention is typically reported as 6 months post-treatment, but may also be reported as 6 months post TQD. Where reported specifically, we extracted data on 6-month post-TQD.

### Search strategy

In consultation with a medical librarian (PR), we established a search strategy. We searched independently, in duplicate, the following 10 databases (from inception to October 1, 2008): MEDLINE, EMBASE, Cochrane CENTRAL, AMED, CINAHL, TOXNET, Development and Reproductive Toxicology, Hazardous Substances Databank, Psych-info and Web of Science, databases that included the full text of journals (*OVID, ScienceDirect*, and *Ingenta*, including articles in full text from approximately 1700 journals since 1993). In addition, we searched the bibliographies of published systematic reviews.[5,19,7,10,11,13,26] and health technology assessments.[27] Searches were not limited by language, sex or age.

### Study selection

Two investigators (EM, PW) working independently, in duplicate, scanned all abstracts and obtained the full text reports of records, that indicated or suggested that the study was a RCT evaluating a smoking abstinence therapy on the outcomes of interest. After obtaining full reports of the candidate trials (either in full peer-reviewed publication or press article) the same reviewers independently assessed eligibility from full text papers.

### Data collection

Two reviewers (PW, EM) conducted data extraction independently using a standardized pre-piloted form. Reviewers collected information about the smoking intervention tested, the population studied (age, sex, underlying conditions), treatment dosages and dosing schedules, the treatment effect at 4 weeks post-TQD and at 6 months post-intervention, the specific measurement of abstinence (sustained or point-prevalence), and the chemical confirmation methods. Study evaluation included general methodological quality features including allocation concealment, sequence generation, blinding status, intention-to-treat, and appropriate descriptions of loss to follow-up. We entered the data into an electronic database such that duplicate entries existed for each study; when the two entries did not match, we resolved differences through discussion and consensus.

### Data analysis

In order to assess inter-rater reliability on inclusion of articles, we calculated the *Phi *statistic, which provides a measure of inter-observer agreement independent of chance.[28] We calculated the Odds Ratios [OR] and appropriate 95% Confidence Intervals [CIs] of outcomes according to the number of events of abstinence reported in the original studies or sub-studies. Odds Ratios are the preferred effect measure in smoking cessation trials. In circumstances of zero outcome events in one arm of a trial, we added 1 to each arm, as suggested by Sheehe.[29] We first pooled studies of all NRT interventions versus all controls using the DerSimonian-Laird random effects method,.[30] which recognizes and anchors studies as a sample of all potential studies, and incorporates an additional between-study component to the estimate of variability.[31] We calculated the I^2 ^statistic for each analysis as a measure of the proportion of the overall variation that is attributable to between-study heterogeneity.[32] Forest plots are displayed for each primary analysis, showing individual study effect measures with 95% CIs, and the overall DerSimmonian-Laird pooled estimate. We then conducted a meta-regression analysis on the NRT studies with predictors of heterogeneity including the following covariates: placebo control; reporting of sequence generation; reporting of allocation concealment; use of gum or patch; and, method of chemical confirmation of abstinence. We additionally conducted separate pooled analyses of NRT versus placebo, gum versus control and patch versus control. We conducted all analyses at 4 weeks and also at 6 months post-TQD. For bupropion trials, we pooled all bupropion trials versus all controls and conducted a meta-regression analysis using the following covariates: placebo control; reporting of sequence generation; reporting of allocation concealment; method of chemical confirmation of abstinence; and plans to quit. We conducted separate meta-regression analyses and calculated the relevant ORs for the covariates as the exponent of the coefficient.[33] We additionally pooled all placebo-controlled trials and evaluated effect sizes at 4 weeks and at 6 months post-TQD. For head-to-head trials of bupropion versus NRT, we conducted pooled random-effects analyses at 4 weeks and at 6 months post-TQD. For varenicline trials, we conducted pooled random-effects analyses of varenicline versus placebo and for head-to-head trials of varenicline versus bupropion or NRT at 4 weeks year and at 6 months. post-TQD. Head-to-head trials provide the strongest inferences regarding intervention superiority.[34] However, with so few head-to-head trials of varenicline versus NRT, we conducted indirect comparisons of these interventions versus placebo using methods described by Bucher et al.[35] This method maintains the randomization from each trial and compares the summary estimates of pooled interventions with CIs. Analyses were conducted using StatsDirect (version 2.5.2, ) and Comprehensive Meta-analysis (version 2, ).

## Results

### Study inclusion

**Figure 1:**
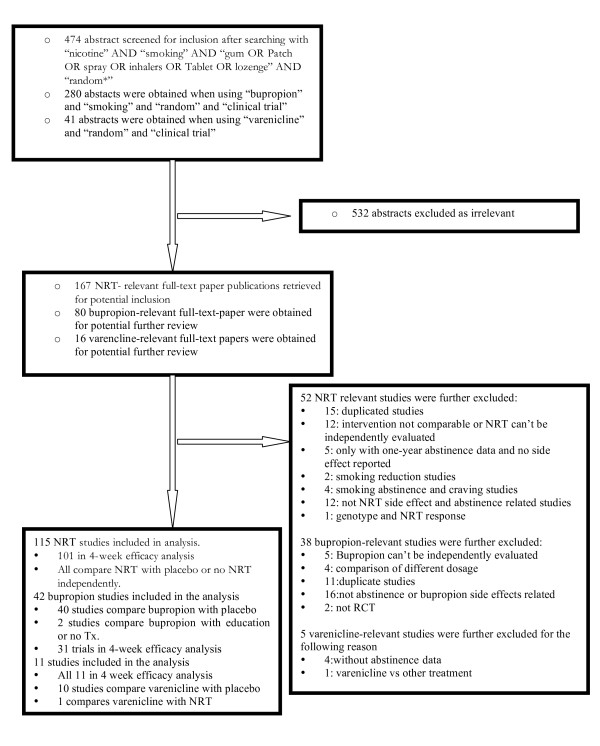
**Flow diagram of included studies**.

We identified 795 abstracts from our extensive searches. We excluded 532 as irrelevant to meeting our inclusion criteria. We obtained 263 full-text studies for screening. We further excluded 94 studies for reasons explained in figure 1 [See Additional File 1]. In total, we included data from 168 RCTs. Agreement was near perfect (φ => 0.9).

### Methods reporting

#### Nicotine Replacement Therapy

One hundred and fifteen RCTs of NRT provided either safety or efficacy data at approximately 4 weeks post-TQD. [36-150]. Eighty-two (82/115) used a placebo control [36-116,150]. Trials were variably reported with only 43 reporting methods of sequence generation[37,39,41,46,52,55,57,70,73-76,80,83,85-92,95-98,103,105,110-112,114-116,118,121,125,126,139,142,144,145,148]. Eighteen (18/115) reported on allocation concealment. [37,39,41,46,70,76,81,84,86,88-90,95,105,111,112,126,148], 81 (81/115) reported on who was blinded[36-73,75,120,131,132-94,96-98,149,100-103,105-116]. Most trials used some form of chemical confirmation of abstinence, with carbon monoxide being the most common (104/115).[36-38,40-57,59-71,73,117-120,122-124,129-134].[72,74-81,83-94,97,135,137,149-111,113-116-148], salivary cotinine (26/115).[42,45,46,50,56,66,68,75,76,79,83,93,95,103,106,111,123,125,128,129,132-134,145,147,150], serum status (7/115).[39,43,58,71,114,119,136], or urine sampling (4/115)[74,112,126,129]. Most (94/115) reported that participants were trying to quit smoking.[36-39,41,44-52,54-65,117,118,121,122,124-129,131,132,68-75,77,78,80-82,85-87,89-91,93,94,97-100,102-106,108,110-116,136-140,143-149].

#### Bupropion

Forty-two bupropion trials met our inclusion criteria.[113,114,142,143,149,151-187] and reported on outcomes at 4 weeks post-TQD. Almost all trials (40/42) used a placebo control.[113,114,149,151-187], with 2 providing education.[143] and counseling.[142] as controls. The quality of reporting studies varied considerably. We found that important study quality indicators were reported sporadically. Sequence generation was reported in 23 of 42 trials.[113,114,142,152-154,157-159,161-164,169-173,176,180,182,185,186], allocation concealment was reported in 12 of 42 trials.[152,153,157-159,162-164,170,176,182,186], the status of who was blinded was reported in 38 of 42 trials.[113,114,142,149,151-174,176,177,180-187], 37 trials.[113,114,142,143,149,151-155,157-165,167-172,174-177,180-187] confirmed cessation using carbon monoxide testing, while 13 used urinary cotinine[114,152,153,157-159,166,173,174,178-180,184]. Almost all trials used participants that were planning to quit smoking (38/42).[113,114,142,143,149,151-161,163,165-171,173-180,182-187].

#### Varenicline

Eleven varenicline studies met our inclusion criteria[162-164,188-195]. One reported only on safety[193]. All trials had a placebo control, 3 also had a bupropion control in their 3 armed trials[163,164,188]. We found that almost all (7/11) provided an additional intervention of counseling available[162-164,190-192,194]. Sequence generation was reported in 6 of 11 studies.[162-164,189,192,195], allocation concealment in 7 of 11 studies.[162-164,189,192,194,195], blinding status in all studies (11/11), and the use of carbon monoxide testing in 10 of 11 studies.[162-164,188-192,194,195], and urinary cotinine in 1 of 11 studies[193]. Five trials reported that the participants were trying to stop smoking[163,189,190,192,195].

### Effectiveness

#### Nicotine Replacement Therapy

**Figure 2:**
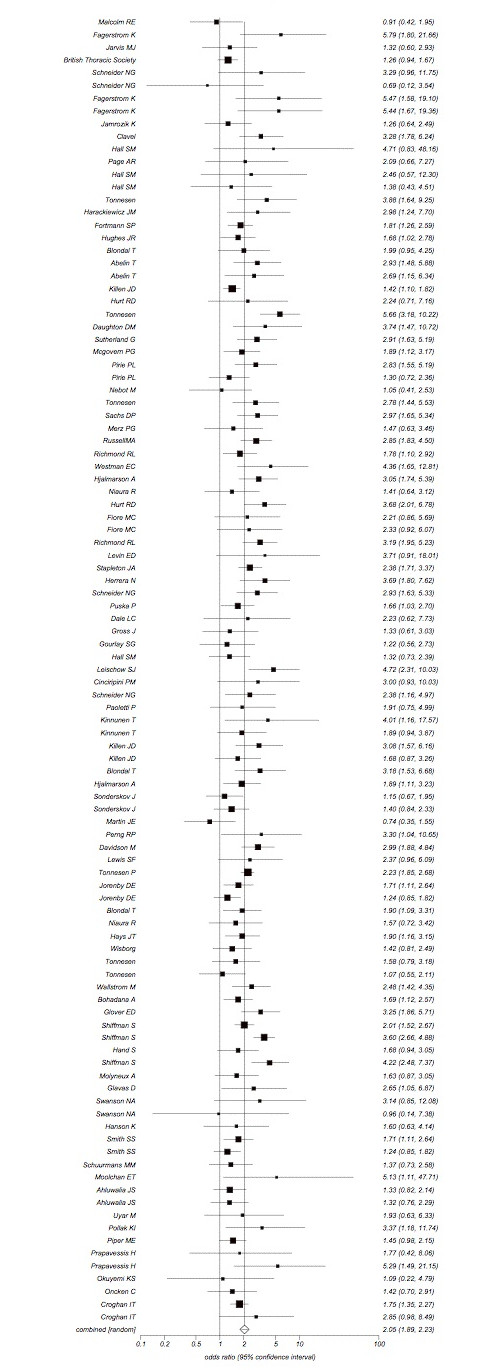
**Random effects meta-analysis of all NRT trials combined versus all inert controls at 4 weeks**. post-TQD.

**Figure 3:**
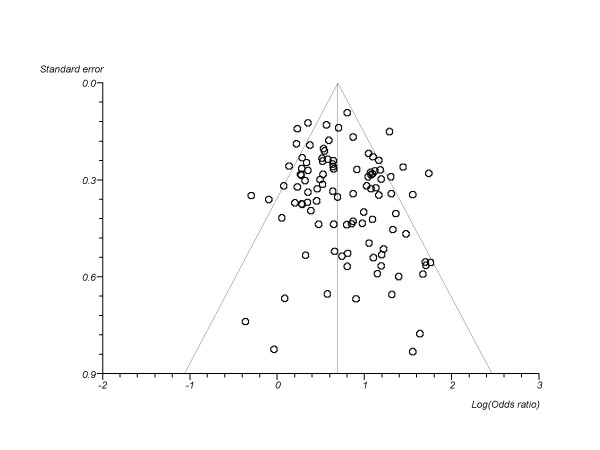
**Funnel plot evaluating publication bias in NRT versus control event rates at 4 weeks post-TQD**.

We combined a total of 101 trials.[36-43,45-47,49-52,54-65,117-119,121,123,128-132,147].[66-69,71,73-82,84,86,134,135,137,149,91,94,95,97-100,103,105,106,111,114-116-146]evaluating some delivery form of NRT versus inert controls at approximately 4 weeks post-TQD (total n = 31,321). The pooled overall OR is OR 2.05 (95% CI, 1.89-2.23, P =< 0.0001, I^2 ^= 51.8%, 95% CI = 38% to 61.3%, P =< 0.0001, See Figure 2). This assessment permitted a sufficient number of studies to assess publication bias and we found marginal evidence of it (Egger's P = 0.055, See Figure 3). We evaluated whether reporting exactly 4 week post-TQD data influenced outcomes and found trials reporting exactly 4 week post-TQD data were more likely to report treatment effects (OR 2.11, 95% CI, 1.97-2.27, P =< 0.0001). These pooled trials yielded an OR 1.82, 95% CI, 1.62-2.05, P =< 0.0001, I^2 ^= 41.6%, 95% CI, 9.1 to 59.1%, P = 0.002). Trials reporting on sustained abstinence at approximately 4 weeks post-TQD yielded a similar pooled estimate (38 RCTs.[45,52,54,56,57,60,61,66,67,69,73,75,81,82,86,87,89,91,94,98,99,103,116,124,129,131,139,142,145,149], n = 17,606, OR 2.24, 95% CI, 1.94-2.28, P =< 0.0001, I^2 ^= 67.7%, 95% CI = 53.7% to 76.1%, P =< 0.0001). When we evaluated trials assessing NRT only to placebo we pooled 74 trials.[36-43,45-47,49-52,54-69,71,73-82,131,84,86-91,94,95,97,98,100,103,105,106,111,114-116,149] (total n = 25,154: 24,654) and found a pooled estimate of 2.13 (95% CI, 1.94-2.34, P =< 0.0001, I^2 ^= 53.6%, 95% CI = 37.6% to 64%, P =< 0.0001, this was not dissimilar when evaluating sustained abstinence (29 RCTs.[45,52,54,56,57,60,61,66,67,69,73,75,81,82,86,87,89,91,94,98,99,103,124,131,149], n = 14,306, OR 2.36 (95% CI, 2.04-2.73 I^2 ^= 61.4%, 95% CI = 37.5% to 73.5%, P =< 0.0001).

When we specifically looked at the effectiveness of NRT gum versus all inert controls we pooled data from 41 trials.[36-42,45-47,50,67,74,78,106,111,114,117-119,121,123,124,128-132,134,137,138,141,144,146] (n = 9,460) and found an OR of 1.76 (95% CI, 1.54-2.01, P =< 0.0001, I^2 ^= 38.9% (95% CI = 3.8% to 57.6%, P = 0.004). This was not dissimilar from gum versus placebo controls (23 trials.[36-42,45-47,50,67,74,78,106,111,114,124,131], n = 5818, OR 1.66, 95% CI, 1.41-1.96, P =< 0.0001, I^2 ^= 41.1% P = 95% CI = 0% to 63.2%, P = 0.01). When we specifically examined trials assessing the effectiveness of NRT cutaneous patches versus inert controls we included data from 47 RCTs.[49,51,52,54,56,58-60,62-66,69,71,73,77,79,82,84,86,87,89-91,95,97,100,103,105,106,115,135,139,141-145,149] (n = 15,980) and found a pooled estimate of 2.11 (95% CI, 1.85-2.40, P =< 0.0001, I^2 ^= 54.8%, 95% CI, 34.7 to 66.7%, P =< 0.0001). This was not different when examining NRT patches versus placebo controls (38 trials [49,51,52,54,56,58-60,62-66,69,71,73,77,79,82,84,86,87,89-91,95,97,100,103,105,106,115,149], n = 14,988, OR 2.15, 95% CI, 1.86-2.48, P =< 0.0001, I^2 ^= 59.5%, 95% CI = 39.3 to 70.8%, P =< 0.0001).

When evaluating NRT versus controls at 6 months (96 RCTs, n = 30,422) we found a pooled estimate of OR 1.92 (95% CI, 1.73-2.14, P =< 0.0001, I^2 ^= 64.2%, 95% CI, 54.8 to 70.8%, P =< 0.0001). This was not dissimilar when evaluating NRT as either gum (23 RCTs, n = 5818, OR 1.69, 95% CI, 1.37-2.08, P =< 0.0001, I^2 ^= 55.9%, 95% CI, 21.8 to 71.3%, P = 0.0004) or cutaneous patch (43 RCTs, n = 16,298, OR, 1.90, 95% CI, 1.62-2.33, I^2 ^= 62.4%, 95% CI, 45.5 to 72.3%, P =< 0.0001).

#### Bupropion

**Figure 4:**
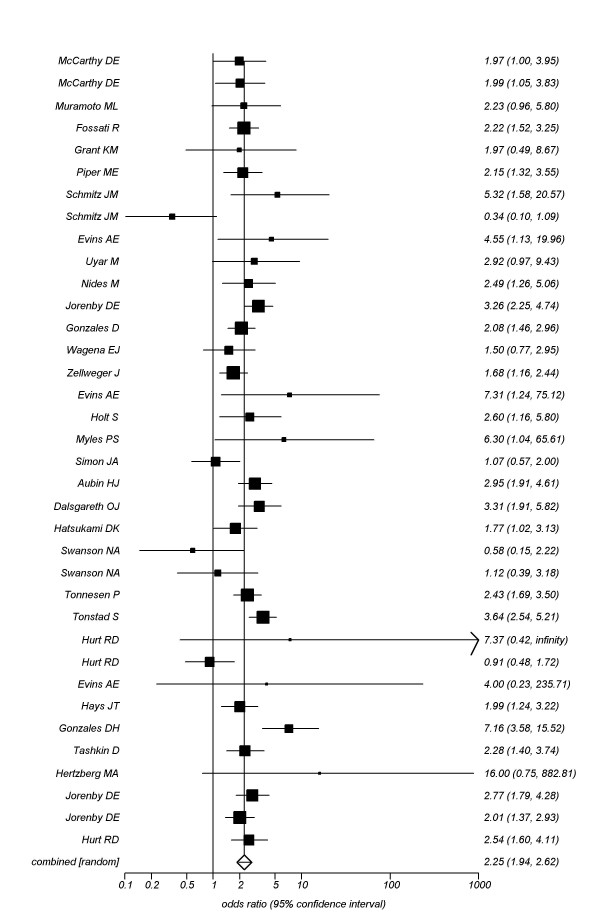
**Random effects meta-analysis of smoking cessation with bupropion versus controls at 4-weeks post-TQD**.

**Figure 5:**
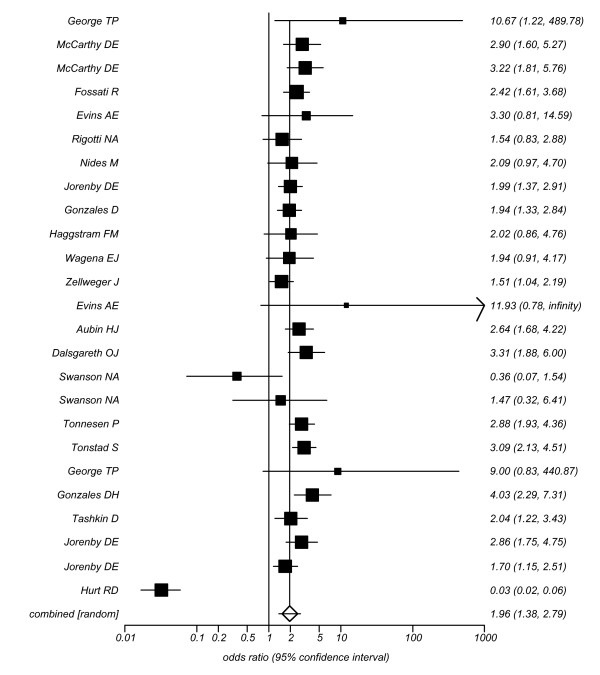
**Random effects meta-analysis of sustained smoking abstinence with bupropion versus controls at 4-weeks post-TQD**.

We pooled data from 31 trials.[114,142,143,149,152-157,162-173,175-177,182-187] contributing a total n of 11,118 participants providing data at approximately 4 weeks post-TQD and found a pooled OR of 2.25 (95% CI, 1.94-2.62, P =< 0.0001, I^2 ^= 78, 95% CI, 70-83%, P =< 0.001, See Figure 4). When we evaluated studies assessing sustained cessation (25 randomized cohorts.[142,149,151,152,154,155,159,160,162-166,168,170,171,175,176,180,182,185,187], n = 8,724) we found a pooled OR of 1.96, 95% CI, 1.39-2.79, P = 0.0002, I^2 ^= 89%, 95% CI, 86-92%, P =< 0.0001, See Figure 5). We were able to explain the large heterogeneity in the analysis through meta-regression as studies failing to report allocation concealment were associated with increased effect sizes (OR 2.29, 95% CI, 2.05-2.60, P =< 0.0001), as were studies confirming abstinence through urinary cotinine (OR 2.44, 95% CI, 2.18-2.66, P =< 0.0001), but not those utilizing carbon monoxide confirmation (OR 1.30, 95% CI, 0.87-1.95, P = 0.18).

Our secondary outcomes for effectiveness also indicated significant benefits with bupropion over controls at 6 months (OR 1.75, 95% CI, 1.54-1.97, P =< 0.0001, I^2 ^= 32%, 95% CI, 0-53%, P =< 0.0001). This effect was consistent when applying only continuous abstinence in the 6 month period (OR 1.94, 95% CI, 1.62-2.32, P =< 0.0001, I^2 ^= 34, 95% CI, 0-62, P = 0.04).

#### Varenicline

**Figure 6:**
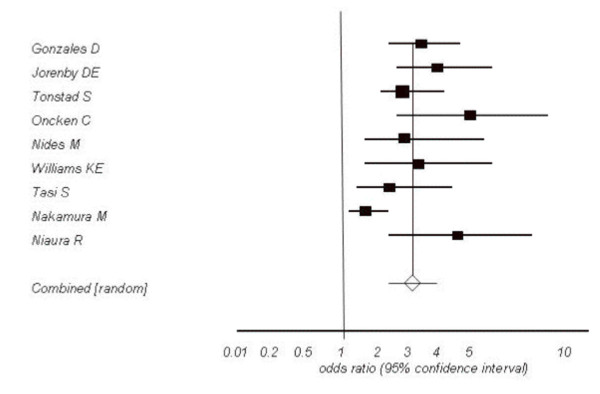
**Random effects meta-analysis of varenicline versus placebo at 4 weeks post-TQD**.

When we evaluated varenicline for smoking abstinence at approximately the last 4 weeks of treatment (4 weeks post-TQD) compared to placebo, we pooled 9 trials.[162-164,189-192,194,196] contributing a total n of 5,192 participants. Our pooled estimate for abstinence at 4 weeks post-TQD found a pooled OR of 3.16 (95% CI, 2.55-3.91, P =< 0.0001, I^2 ^= 53%, 95% CI, 0-76%, P = 0.02, See Figure 6). We were able to explain the heterogeneity in the analysis through meta-regression as studies failing to report allocation concealment were associated with increased effect sizes (OR 3.35, 95% CI, 2.45-4.57, P =< 0.0001). Our 6 month evaluations of varenicline versus placebo yielded similar estimates for continuous abstinence in the 6 month period (OR 2.17, 1.48-3.19, P =< 0.0001, I^2 ^= 80, 95% CI, 49-90%, P =< 0.0001).

Two trials evaluated head to head comparison of varenicline and bupropion and found a pooled estimate of OR 1.86 (95% CI, 1.49-2.33, P =< 0.0001) using continuous abstinence rates at 4 weeks and, at 6 months post-TQD (OR 1.64, 95% CI, 1.28-2.10, P =< 0.0001).[163,164] One trial evaluated varenicline versus NRT patch (n = 757) for continuous abstinence at the last 4 weeks post-TQD using carbon monoxide confirmation (OR 1.70, 95% CI, 1.26-2.28, P =< 0.001).[188] This same trial reported on continuous abstinence at 6 months (24 weeks), but the difference was not significant (OR 1.29, 95% CI, 0.94-1.77, P = 0.11).

#### Adjusted indirect comparison (Figure 7)

**Figure 7:**
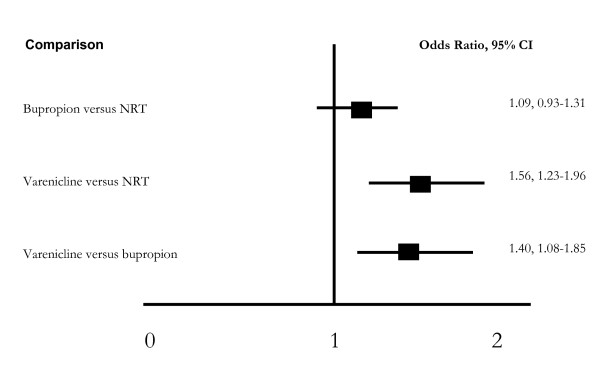
**Indirect comparisons**.

We applied an adjusted indirect comparison evaluating NRT, bupropion and varenicline on our primary endpoint of 4 weeks post-TQD abstinence. We were unable to display a significant difference between NRT and bupropion at 4-weeks (OR, 1.09, 95% CI, 0.93-1.31, P = 0.28). Varenicline was superior to both NRT (OR 1.56, 95% CI, 1.23-1.96, P = 0.0002) and bupropion at post-TQD (OR 1.40, 95% CI, 1.08-1.85, P = 0.01).

## Discussion

This study confirms the short-term effectiveness of all three smoking interventions compared to placebo. Our findings stand in line with outcomes evaluated over a longer period, up to one year, of these same interventions.[9,10] This finding should be of interest to clinicians, policy-makers and patients. As interventions to assist in smoking cessation are increasingly available, the combination of these interventions, along with socio-behavioural interventions, should be a research priority.[8]

The definition of smoking abstinence and relapse are variable across studies. The most common time periods of smoking cessation required to be considered abstinent are 24 hours, 7 days and 30 days. Relapse is defined by the National Heart, Lung and Blood Institute as having smoked at least a puff for 7 days after having quit. Seventy five to 80 percent of smokers relapse within the first 6 months. Relapse rates continue to remain high from 6 to 12 months (7 to 35% of those abstinent at 6 months). Relapse occurs at a lower rate following one year of cessation.[4] The National Center for Health Education Code of Practice and Standards for the Evaluation of Group Smoking Cessation Programs recommends at least one year of follow-up before determining if patients have quit smoking.[4] The National Institute for Clinical Excellence (UK) Guidelines require the reporting of short-term abstinence rates. Further, immediate abstinence of smoking following a major cardiovascular event has major benefits in preventing secondary events.[197] We recognize that multiple short-term abstinence attempts followed by relapses may be associated with long term smoking use, an issue that is increasingly complex to manage from a clinical and public health perspective.[198] However, our findings are consistent with the longer term evaluations and indicate that sustained abstinence is possible in the clinical trial setting. Furthermore there are some physiological and health advantages to short-term abstinence. For example, individuals with cardiovascular events can immediately benefit from smoking discontinuation because of improvements in several physiological variables including reduced myocardial oxygen demand, improved myocardial oxygen supply, reduced activation of the sympathetic system, reduced risk of arrhythmias and reduced acute thrombosis risk. These benefits could be particularly critical in the peri-event period when patients are at increased risk of complications or repeat events. Thus even if relapse occurs at a later stage, abstinence around the time of an event could prove beneficial.

When we previously evaluated varenicline to NRT and bupropion, we had data from only 4 trials.[9] This evaluation found that the addition of 7 trials continues to demonstrate elevated varenicline effects compared to NRT and bupropion. Further community effectiveness interventions will be required to ensure generalizability.

There are several strengths and limitations to consider when interpreting our analysis. Strengths of this review include the comprehensive search strategy that improved the likelihood of identifying all relevant studies. Duplicate extraction of data reduced the potential for bias in this component of the synthesis process. By limiting this review to randomized trials we ensured that the included studies would have reduced likelihood of systematic error and therefore have high internal validity. Our use of meta-regression to identify sources of heterogeneity in the meta-analyses is a strength and demonstrated that several of the *a priori *chosen covariates were predictors of heterogeneity. To reduce patient-reporting bias, we included only studies that chemically confirmed the cessation of smoking at the specific time-points- this has been a weakness in previous reviews.[23]

Limitations of this meta-analysis include the potential for publication bias, in particular the possibility that small negative studies would not be published. Publication bias on short-term effects is likely due to both author-initiated bias and journal-initiated bias against short-term evaluations. We included only published trials so it is possible that other trials have been conducted and never published. However, it is unlikely that the presence of these studies would have altered the findings of our analysis given the large number of studies included and the consistency with the longer-term evaluations (both 6 months and one year).[9,10] We limited our search to English language databases (although we would include non-English articles if identified) so the possibility of quality studies in other languages does exist. We used both direct and indirect comparisons to evaluate the relative effectiveness of agents. Head-to-head trials provide the strongest inferences regarding intervention superiority.[34] In the presence of existing head-to-head trials of varenicline versus NRT.[188] and bupropion,.[163,164] it is arguable whether indirect comparisons are required.[199] In this case, the results were consistent. We used the indirect comparison method proposed by Bucher et al., that respects the principle of randomization between trials.[200] Other strategies we have previously applied,.[201] including mixed treatment comparisons, offer similar benefits.[199]

## Conclusion

In conclusion, our review demonstrates clear efficacy of smoking cessation pharmacotherapies in the short term and provides similar estimates of efficacy as longer term evaluations.[9,10] Given the benefits of smoking abstinence in both primary and secondary prevention of major morbidities, the use of these therapies in patients with active smoking related disease warrants further study.[15] Future research to evaluate the efficacy and safety of these interventions in combination and in patients with advanced diseases is warranted.

## Abbreviations

CO: Carbon monoxide; NRT: Nicotine replacement therapy; OR: Odds ratio; RCT: Randomized Clinical Trial; SCN: Saliva thiocynate; 95% CI: 95% Confidence intervals.

## Competing interests

EM, PW and KW have consulted to Pfizer Ltd in the past 5 years. No stock ownership is reported. DS is an employee of Pfizer Ltd. JE declares no conflict of interest. Pfizer Ltd. Is the maker of an NRT product and varenicline. EM and KW are supported by Canadian Institutes of Health Research (CIHR) Canada Research Chairs.

## Authors' contributions

EM, PW, DS, KW and COR conceived the protocol. EM, PW, KW did the search strategies. EM, PW, JO, KW did the data abstraction and analysis. EM, PW, JO, KW wrote the first draft of the manuscript. EM, PW, DS, JO, KW approved the final submitted version.

## Funding

This study received unrestricted funding from Pfizer Ltd to evaluate anti-smoking agents. They had no role in the conduct, interpretation or writing of this manuscript.

## Supplementary Material

Additional file 1 **Characteristics of included studies**. Supplementary Tables addressing study populations and interventions. Click here for file
