# Supplementary material for: Efficacy of pharmacotherapies for short-term smoking abstinance: A systematic review and meta-analysis
Source: Harm Reduct J. 2009 Sep 18;6:25. doi: 10.1186/1477-7517-6-25 (PMC2760513; doi:10.1186/1477-7517-6-25)
Supplement: Additional file 1 — Characteristics of included studies. Supplementary Tables addressing study populations and interventions. [file 1477-7517-6-25-S1.DOC]

| year | author | country | Char. Of the participant | cig/day, *(mean, median) | smoking year *(mean, median) | Intervention 1.gum  2.Patch 3.nasalspray 4.inhalers 5.Tablet 6.lozenge | dosage (mg) | **Tx。and the number in each group** | | | | co-intervention | duration of the treatment |
| --- | --- | --- | --- | --- | --- | --- | --- | --- | --- | --- | --- | --- | --- |
|  |  |  |  |  |  |  |  | intervention | number | control | number |  |  |
| 1980 | Malcolm RE | England | healthy | 25* | na | 1 | 2 | gum | 73 | placebo | 63 | no | 3mon |
| 1982 | Fagerstrom K | Sweden | healthy | na | na | 1 | 2 | gum | 47 | placebo | 49 | psychological treatment | 4wks |
| 1982 | Jarvis MJ | England | healthy | >20 | na | 1 | 2 | gum | 58 | placebo | 58 | group counseling | 12mon |
| 1983 | British Thoracic Society | England | healthy | >=1 | na | 1 | 2 | gum | 410 | placebo | 412 | verbal advice+ booklet | 3-6mon |
|  |  |  |  |  |  |  |  |  |  | no tx. | 401 |  |  |
| 1983 | Russell MA | England | healthy | 17.5* | na | 1 | 2 | gum | 679 | booklet | 675 | advice | na |
| 1983 | Schneider NG | USA | healthy | >=20 | na | 1 | 2 | gum | 30 | placebo | 30 | clinic-support | na |
|  |  |  |  |  |  |  |  |  | 13 |  | 23 | minimal intervention |  |
| 1984 | Fagerstrom K | Sweden | healthy | 19* | na | 1 | 2 to 4 | gum | 50 | no Tx. | 22 | long follow-up | 3mon |
|  |  |  |  |  |  |  |  |  | 46 |  | 27 | short follow up |  |
| 1984 | Jamrozik K | England | healthy | na | na | 1 | 2 | gum | 101 | placebo | 99 | no | 3mon |
| 1985 | Clavel | France | healthy | >=5 | na | 1 | 2 | gum | 205 | no Tx. | 222 | no | 105pieces |
| 1985 | Hall SM | USA | healthy | 30.5* | na | 1 | 2 | gum | 35 | no Tx. | 36 | intensive behavioral therapy | 6mon |
| 1986 | Page AR | Canada | healthy | 18.8* | na | 1 | 2 | gum | 96 | no tx. | 114 | advice | 3mon |
| 1987 | Hall SM | USA | healthy | 30* | na | 1 | 2 | gum | 36 | placebo+ | 34 | low contact condition | 1y |
|  |  |  |  |  |  |  |  |  | 35 |  | 34 | behavioral Tx. |  |
| 1988 | Sutton | England | healthy | 19* | na | 1 | 2 | gum | 32 | no tx. | 82 | counseling | 105p |
| 1988 | Tonnesen | Sweden | healthy | >=10 | na | 1 | 2 | gum | 60 | placebo | 53 | counseling | 2-24mon |
| 1988 | Harackiewicz JM | USA | healthy | 26.5* | 17* | 1 | 2 | gum | 99 | no Tx. | 52 | self-help manual | 6mon |
| 1988 | Tonnesen P | Denmark | healthy | >=10 | 10-65 | 1 | 2,4 | gum | 116 | advice | 56 | no | >=6wks |
| 1988 | Areechon W | Sweden | healthy | >=15 | 24* | 1 | 2 | gum | 98 | placebo | 101 | lecture | 3mon |
| 1988 | Fortmann SP | USA | healthy | 24* | 25* | 1 | 2 | gum | 299 | placebo | 148 | no | 3mon |
|  |  |  |  |  |  |  |  |  |  | no Tx. | 153 |  |  |
| 1989 | Hughes JR | USA | healthy | 30* | 19* | 1 | 2 | gum | 210 | placebo | 105 | brief advice | 3mon |
| 1989 | Gilbert RJ | Canada | primary care patient | >=1 | na | 1 | 2 | gum | 112 | no tx. | 111 | supportive visit | 2-3mon |
| 1989 | Blondal T | Iceland | healthy | 21g * | na | 1 | 4 | gum | 92 | placebo | 90 | education session | 3mon |
| 1989 | Gross J | USA | healthy | >=10 | 24* | 1 | na | gum | 20 | placebo | 20 | no | 10wks |
| 1989 | Abelin T | Switzerland | healthy | >20 | 21* | 2 | 7 to 21 | patch | 100 | placebo | 99 | no | 12wks |
|  |  |  |  |  | 22* | 2 |  |  | 56 |  | 56 |  | 9wks |
| 1990 | Killen JD | USA | healthy | 24* | 24* | 1 | 2 | gum | 600 | placebo | 309 | self-guided behavioral treatment | 8wks |
|  |  |  |  |  |  |  |  |  |  | no tx. | 309 |  |  |
| 1990 | Hurt RD | USA | healthy | >=20 | >=1 | 2 | 21 | patch | 31 | placebo | 31 | no | 6wks |
| 1991 | Segnan N | Italy | healthy | na | na | 1 | na | gum | 294 | no tx. | 275 | repeated counseling | 3mon |
| 1991 | Tonnesen | Sweden | healthy | >=10 | >=3 | 2 | 15 | patch | 145 | placebo | 144 | psychological support | 12wks |
| 1991 | Campbell IA | England | hospitalized patients | na | na | 1 | 4 | gum | 107 | placebo | 105 | advice | 3mon |
| 1991 | Uckene JK | USA | healthy | 23* | 16* | 1 | 2to4 | gum | 402 | no tx. | 420 | counseling | 12wks |
| 1991 | Daughton DM | USA | healthy | >=20 | 23.9* | 2 | na | patch(24hr) | 51 | placebo | 52 | no | 4wks |
|  |  |  |  |  |  |  |  | patch(wakeful hour) | 55 |  |  |  |  |
| 1992 | Sutherland G | England | healthy | 25* | 22* | 3 | 1 | spray | 116 | placebo | 111 | no | 3mon |
| 1992 | Mcgovern PG | USA | healthy | >=25 (58% people) | na | 1 | 2 | gum | 146 | no tx. | 127 | education program | 3mon |
| 1992 | Pirie PL | USA | healthy women | 25* | na | 1 | 2 | gum | 108 | No Tx. | 103 | smoking clinic program | 2-5mon |
|  |  |  |  |  |  |  |  |  | 98 |  | 108 | smoking clinic and weight control program |  |
| 1992 | Nebot M | Spain | healthy | >=15 | na | 1 | 2 | gum | 93 | no Tx. | 175 | physician counseling | 2-4wks |
| 1993 | Tonnesen | Sweden | healthy | >=10 | >=3 | 4 | 0.1umol/per puff | inhaler | 145 | placebo | 141 | no | 3-6mon |
| 1993 | Sachs DP | USA | healthy | >=10 | >=3 | 2 | 15 | patch | 113 | placebo | 107 | physician counseling | 12-18wks |
| 1993 | Merz PG | Germany | healthy | >=20 | >=1 | 2 | 7 to 21 | patch | 80 | placebo | 80 | no | 3mon |
| 1993 | RussellMA | 15 English counties | healthy | >=15 | na | 2 | 5 to 15 | patch | 400 | placebo | 200 | no | 18wks |
| 1993 | Richmond RL | Australia | healthy | na | na | 1 | na | gum | 200 | no tx. | 150 | structured behavioral change | 3mon |
| 1993 | Westman EC | USA | healthy | >=20 | 22* | 2 | 12.5 to 25 | patch | 79 | placebo | 80 | counseling | 6wks |
| 1994 | Flowler G | England | general practice patients | na | na | 2 | na | patch | 842 | placebo | 844 | no | 12wks |
| 1994 | Hjalmarson A | Sweden | healthy | 21* | 26* | 3 | 1 | spray | 125 | placebo | 123 | na | 3mon |
| 1994 | Niaura R | USA | healthy | 29* | 24* | 1 | 2 | gum | 84 | no Tx | 89 | self-help treatment | 1-4mon |
| 1994 | Hurt RD | USA | healthy | >=20 | past 1 year | 2 | 22 | patch | 120 | placebo | 120 | individual counseling | 8wks |
| 1994 | Fiore MC | Ireland | healthy | >=15 | >=1 | 2 | 22 | patch | 44 | placebo | 43 | group counseling | 8wks |
|  |  |  |  |  |  |  | 11 to 22 |  | 57 |  | 55 | individual counseling | 6wks |
| 1994 | Richmond RL | Australia | healthy | >20 | 24* | 2 | 7 to 21 | patch | 158 | placebo | 157 | behavioral therapy | 10wks |
| 1994 | Levin ED | USA | healthy | 28* | 23* | 2 | 22 | patch | 31 | placebo | 31 | group counseling | 8wks |
| 1995 | Stapleton JA | England | healthy | >=15 | na | 2 | 15 | patch | 400 | placebo | 400 | Booklet + advice | 18wks |
|  |  |  |  |  |  |  | 25 |  | 400 |  |  |  |  |
| 1995 | Fortmann SP | USA | healthy | >=25 | na | 1 | 2 | gum | 262 | no Tx. | 261 | no | na |
|  |  |  |  |  |  |  |  |  | 260 |  | 261 | self-help material |  |
| 1995 | Herrera N | Sweden | healthy | >=10 | na | 1 | 2 | gum | 76 | placebo | 78 | behavior modification program | 3mon |
| 1995 | Schneider NG | USA | healthy | >=15 | 22* | 3 | 1 | spray | 128 | placebo | 127 | no | 6wks-6mon |
| 1995 | Puska P | Finland | healthy | >=10 | >=3 | 2 | 15 | patch | 150 | placebo | 150 | gum | 12-18wks |
| 1995 | Kornitzer M | Sweden | healthy | >=10 | na | 1 | 2 | gum | 149 | placebo | 150 | patch | 12-24wks |
|  |  |  |  |  |  | 2 | 5to15 | patch | 150 | placebo | 75 | placebo gum |  |
| 1995 | Dale LC | USA | healthy | >=10 | >=1 | 2 | 11 | patch | 18 | placebo | 18 | no | 8wks |
|  |  |  |  |  |  |  | 22 |  | 17 |  |  |  |  |
|  |  |  |  |  |  |  | 44 |  | 18 |  |  |  |  |
| 1995 | Gross J | USA | healthy | 33* | na | 1 | 2 | gum | 131 | no tx. | 46 | no | 3mon |
| 1995 | Gourlay SG | Australia | healthy | >=15 | 23* | 2 | 7 to 21 | patch | 315 | placebo | 314 | behavioral counseling | 3mon |
| 1996 | Campbell IA | England | smoking related patient | >=1 | past 1 wk | 2 | 7 to 21 | patch | 115 | placebo | 119 | no | 12 week |
| 1996 | Hall SM | USA | healthy | >=10 | 21* | 1 | 2 | gum | 98 | placebo | 103 | mood management | 12wks |
| 1996 | Leischow SJ | USA | healthy | >=10 | 25* | 4 | na | inhaler | 111 | placebo | 111 | advice | 3-6mon |
| 1996 | Cinciripini PM | USA | healthy | >=15 | >=3 | 2 | 7 to 14 | patch | 32 | no Tx. | 32 | behavior therapy | 9wks |
| 1996 | Schneider NG | USA | healthy | 26* | 25* | 4 | 13ug/per puff | inhaler | 112 | placebo | 111 | behavior intervention | 6mon |
| 1996 | Paoletti P | Italy | healthy | 23* | 21* | 2 | 15 | patch | 60 | placebo | 60 | no | 12wks |
| 1996 | Kinnunen T | USA | depress | 22* | 23.1* | 1 | 2 to 4 | gum | 59 | placebo | 33 | no | 3mon |
|  |  |  | non-depress |  |  |  |  | gum | 119 | placebo | 58 |  |  |
| 1996 | Nilsson P | Sweden | healthy | >10 | >10 | 1.2 | na | NRT | 200 | no Tx. | 171 | supportive group sessions | 4mon |
| 1997 | Killen JD | USA | healthy | >=10 | na | 2 | 7 to 21 | patch | 103 | placebo | 104 | manual | 16wks |
|  |  |  |  |  |  |  |  |  | 109 |  | 108 | manual +video |  |
| 1997 | Blondal T | Sweden | healthy | >=1 | na | 3 | 1 | spray | 79 | placebo | 78 | no | 3mon |
| 1997 | Hjalmarson A | USA | healthy | >=10 | >=3 | 4 | 13 ng/puff | inhaler | 123 | placebo | 124 | behavior modification program | 3-6mon |
| 1997 | Sonderskov J | Denmark | healthy | <20 | na | 2 | 14 | patch | 119 | placebo | 125 | no | 12wks |
|  |  |  |  | >=20 |  |  | 21 |  | 132 |  | 142 |  |  |
| 1997 | Martin JE | USA | recovering alcoholics | 26.8* | 24.4 | 1 | 2 | gum | 63 | physical exercise | 72 | behavioral counseling | 4wks |
| 1998 | Daughton D | USA | healthy | >=20 | 19.3* | 2 | 7to21 | patch | 184 | placebo | 185 | counseling | 10wks |
| 1998 | Perng RP | Taiwan | healthy | >=20 | 33* | 2 | 30 | patch | 30 | placebo | 32 | no | 6wks |
| 1998 | Davidson M | USA | healthy | >=20 | >=1 | 2 | 30 | patch | 401 | placebo | 401 | no | 6wks |
| 1998 | Lewis SF | USA | hospitalized patients | >=10 | >=1 | 1 | 11 to 22 | patch | 62 | placebo | 62 | counseling | 6wks |
| 1998 | Ahluwalia JS | USA | healthy | >=10 | >1 | 2 | 7to21 | patch | 205 | placebo | 205 | no | 10wks |
| 1999 | Tonnesen | Europe | healthy | >=14 | >=3 | 2 | 15 | patch | 716 | placebo | 714 | advice brochure | 8 wks |
|  |  |  |  |  |  |  |  |  | 715 |  |  |  | 22wks |
|  |  |  |  |  |  |  | 25 | patch | 715 |  |  |  | 8 wks |
|  |  |  |  |  |  |  |  |  | 715 |  |  |  | 22wks |
| 1999 | Jorenby DE | USA | healthy | 25* | 25* | 2 | 7 to 21 | patch | 244 | placebo | 160 | no | 8wks |
|  |  |  |  |  |  |  |  |  | 245 |  | 244 | bupropion |  |
| 1999 | Blondal T | Sweden | healthy | 25* | >=3 | 3 | 0.5 | spray | 120 | placebo | 119 | nicotine patch | 1y |
| 1999 | Niaura R | USA | healthy | 27.8* | 26.9* | 1 | 2 | gum | 35 | no Tx. | 32 | behavior program | 2mon |
|  |  |  |  |  |  |  |  |  | 31 |  | 31 | behavior+ cue exposure |  |
| 1999 | Hays JT | USA | healthy | >=15 | >=1 | 2 | 22 | patch | 321 | placebo | 322 | no | 6wks |
| 2000 | Wisborg | Denmark | pregnant (>22wks) | >=10 | na | 2 | 10 to 15 | patch | 124 | placebo | 126 | counseling | 11wks |
| 2000 | Tonnesen | Denmark | lung clinic patient | >=10 | na | 2 | 15 | patch | 115 | no tx. | 118 | inhaler | 3-9mon |
|  |  |  |  |  |  | 4 | 13ug/per puff | inhaler |  | no tx. | 104 | patch |  |
| 2000 | Wallstrom M | Sweden | healthy | >=10 | 26* | 5 | 2 | tablet | 123 | placebo | 124 | no | 3-6mon |
| 2000 | Bohadana A | France | healthy | >=10 | >=3 | 2 | 15 | patch | 200 | placebo | 200 | nicotine inhaler | 6wks |
| 2000 | Bolliger CT | Switzerland | healthy | >=15 | >=3 | 4 | 13ug/per puff | inhaler | 200 | placebo | 200 | no | 4mon |
| 2000 | Garvey AJ | USA | healthy | 5 | na | 1 | 2 | gum | 202 | placebo | 203 | counseling | 2mon |
|  |  |  |  |  |  |  | 4 | gum | 203 |  |  |  |  |
| 2002 | Glover ED | USA | healthy | >=10 | >= 3 | 5 | 2 | tablet | 120 | placebo | 121 | no | 3-6mon |
| 2002 | Shiffman S | USA England | healthy TTFC<30min | 17* | na | 6 | 2 | lozenge | 459 | placebo | 458 | behavior support | 6mon |
|  |  |  | healthy TTFC>30min |  |  |  | 4 |  | 450 |  | 451 |  |  |
| 2002 | Hand S | England | healthy | >=1 | na | 2.4 | 10 to 30 | NRT | 136 | no Tx. | 109 | advice & support | 3wks |
| 2002 | Etter J | Switzerland | healthy | >=20 | 3 | 1,2,3 | 2,15,0.5,10,2 | NRT | 265 | placebo | 269 | no | 6mon |
|  |  |  |  |  |  |  |  |  |  | no tx. | 389 |  |  |
| 2002 | Shiffman S | USA | healthy | 25* | 24* | 2 | 7 to 21 | patch | 283 | placebo | 284 | no | 10wks |
| 2003 | Molyneux A | England | hospitalized patients | 20* | 33* | 1 in 5(all) | 2,15,0.5,10,2 | NRT | 91 | no tx. | 91 | counseling | 6wks |
| 2003 | Wennike P | Sweden | healthy | >=15 | >=3 | 1 | 2 | gum | 65 | placebo | 68 | no | 12mon |
|  |  |  |  |  |  |  | 4 | gum | 140 | placebo | 138 |  |  |
| 2003 | Glavas D | Croatia | healthy | >=1 | >=1 | 2 | 7 to 21 | patch | 56 | placebo | 56 | no | 3wks |
| 2003 | Swanson NA | USA | healthy | 19* | 10* | 2 | na | patch | 30 | counseling | 50 | no | 9wks |
|  |  |  |  |  |  |  |  | patch+  bupropion | 30 | no tx.+ bupropion | 30 |  |  |
| 2003 | Hughes JR | USA | alcoholism | >=20 | na | 2 | 7 to 21 | patch | 61 | placebo | 54 | behavioral therapy | 12wks |
| 2003 | Hanson K | USA | adolescence | >=15 | >0.5 | 2 | 7 to 21 | patch | 50 | placebo | 50 | cognitive behavior therapy | 10wks |
| 2003 | Smith SS | USA | healthy | >=15 | >=1 | 2 | 7 to 21 | patch | 244 | placebo | 160 | behavioral treatment | 8wks |
|  |  |  |  |  |  |  |  | patch+ bupropion | 245 | placebo+ burpropion | 244 |  |  |
| 2004 | Chou K | Taiwan | schizophrenia | >=15 | >=1 | 2 | 7to14 | patch | 26 | no Tx. | 42 | no | 8wks |
| 2004 | Schuurmans MM | South Africa | healthy | >=15 | >=3 | 2 | na | patch | 100 | placebo | 100 | no | 2wks |
| 2005 | Batra A | Europe | healthy | >=20 | >=3 | 1 | 4 | gum | 184 | placebo | 180 | no | 12mon |
| 2005 | Moolchan ET | Canada | adolescence | >=10 | >=0.5 | 1 | 2 to 4 | gum | 46 | placebo | 40 | cognitive behavior therapy | 12wks |
|  |  |  |  |  |  | 2 | 14to21 | patch | 34 |  |  |  |  |
| 2005 | Cooper TV | USA | healthy | >=10 | 19 | 1 | 2 | gum | 146 | placebo | 148 | cognitive behavior therapy | 12wks |
| 2006 | Rennard SI | USA | healthy | >=20 | >=3 | 4 | 10 | inhaler | 215 | placebo | 214 | no | 12mon |
| 2006 | Tonnesen P | Sweden | COPD | 20* | na | 5 | 2 | tablet | 95 | placebo | 88 | low support | 12wks |
|  |  |  |  |  |  |  |  |  | 90 |  | 97 | high support |  |
| 2006 | Hotham ED | Australia | pregnant (12-28wks) | >=15 | na | 1 | 15 | patch | 20 | no Tx. | 20 | counseling | 12wks |
| 2006 | Ahluwalia JS | USA | healthy | <10 | >0.5 | 1 | 2 | gun | 189 | placebo | 188 | health education | 8wks |
|  |  |  |  |  |  |  |  |  | 189 |  | 189 | motivational interview |  |
| 2007 | Uyar M | Turkey | healthy | >=10 | >1 | 2 | 7to21 | patch | 50 | advice | 31 | no | 6wks |
| 2007 | Myung SK | Korea | healthy | 15* | 16.5* | 2 | 7to21 | patch | 59 | placebo | 59 | behavioral counseling | 6wks |
| 2007 | Covey LS | USA | healthy | >=10 | na | 2 | 7 to 21 | gum+  bupropion | 74 | placebo+ bupropion | 74 | counseling | 16wks |
|  |  |  |  |  |  |  |  | gum | 73 | placebo | 73 |  |  |
| 2007 | Pollak KI | USA | pregnant (12-25wks) | >=100(lifetime) | na | 1.2.6 | 2,15,0.5,10,2 | NRT | 122 | no tx. | 59 | behavioral therapy | 6wks |
| 2007 | Piper ME | USA |  | >=10 | na | 1 | 2 | gum + bupropion | 228 | placebo+ bupropion | 224 | counseling | 9wks |
| 2007 | Prapavessis H | Canada | woman | >10 | >=3 | 1 | 7 to 21 | patch | 33 | no tx. | 35 | exercise | 6wks |
|  |  |  |  |  |  |  |  |  | 26 |  | 27 | cognitive behavior therapy |  |
| 2007 | Okuyemi KS | USA | healthy | 16* | na | 1 | 4 | gum | 66 | no tx. | 107 | education material | 8wks |
| 2007 | Oncken C | USA | postmenopausal women | >=10 | 33* | 2 | 21 | patch | 57 | placebo | 95 | group counseling | 12wks |
| 2007 | Croghan IT | USA | healthy | >10 | >1 | 4 | na | Inhaler+  bupropion | 567 | no tx. burpropion | 567 | counseling | 3mon |
|  |  |  |  |  |  |  |  | inhaler | 37 | placebo | 37 |  | 3mon |
| 2007 | Gallagher SM | USA | schizophrenia | >=10 | >=3 | 2 | 21 | patch | 60 | no tx. | 60 | contingent reinforcement | 16wks |

**Supplementary Table 1. Characteristics of NRT trials.**

| **Author** | **Year** | **Country** | **Participant Characteristics** | **Cigarettes/day of the participants** | **Pack years** | **Bupropion Dosage (Mg/d)** | **N in Bupropion group** | **Tr. in Control group** | **N in control group** | **Co-treatment in both group** |
| --- | --- | --- | --- | --- | --- | --- | --- | --- | --- | --- |
|  |  |  |  |  |  |  |  |  |  |  |
|  |  |  |  |  |  |  |  |  |  |  |
| George TP | 2008 | USA | schizophrenia | ≧10 | 22 | 300 | 29 | placebo | 29 | Nicotine patch +behavioral therapy |
| McCarthy DE | 2008 | USA | healthy | ≧10 | NA | 300 | 116 | placebo | 113 | No counseling |
|  |  |  |  |  |  | 300 | 113 | Placebo | 121 | counseling |
| Muramoto ML | 2007 | USA | adolescent | ≧6 | NA | 150 | 105 | placebo | 103 | brief individual counseling |
|  |  |  |  |  |  | 300 | 104 |  |  |  |
| Fossati R | 2007 | Italy | healthy | ≧10 | Past 1 yr | 300 | 400 | placebo | 193 | counseling |
| Grant KM | 2007 | USA | alcoholics | ≧20 | NA | 300 | 30 | placebo | 28 | patch |
| Piper ME | 2007 | USA | healthy | ≧10 | NA | 300 | 224 | placebo | 156 | placebo gum |
| Brown RA | 2007 | USA | with depression vulnerability factors | ≧10 | Past 1 yr | 300 | 147 | placebo | 157 | standard treatment |
|  |  |  |  |  |  | 300 | 108 | placebo | 112 | cognitive-behavioral therapy |
| Schmitz JM | 2007 | USA | healthy women | ≧10 | 27 | 300 | 41 | placebo | 39 | cognitive-behavioral therapy |
|  |  |  |  |  |  | 300 | 37 | placebo | 37 | supportive therapy |
| Evins AE | 2007 | USA | schizophrenia | ≧10 | Past 1 yr | 300 | 25 | placebo | 26 | NRT |
| Covey LS | 2007 | USA | healthy | ≧10 | NA | 300 | 74 | placebo | 73 | nicotine gum |
|  |  |  |  |  |  | 300 | 74 | placebo | 73 | placebo gum |
| Uyar M | 2007 | Turkey | Pulmonary disease | ≧10 | ≧1 | 300 | 50 | education | 31 | no |
| Rigotti NA | 2006 | USA | CVD | >1 | past 1 mon | 300 | 124 | placebo | 124 | counseling |
| Nides M | 2006 | USA | healthy | >10 | 24 | 300 | 126 | Placebo | 123 | counseling |
| Jorenby DE | 2006 | USA | healthy | ≧10 | 25 | 300 | 342 | Placebo | 341 | counseling |
| Gonzales DH | 2006 | USA | healthy | ≧10 | 24 | 300 | 329 | Placebo | 344 | counseling |
| Haggstram FM | 2006 | Brazil | healthy | NA | ≧10 | 300 | 53 | placebo | 51 | cognitive-behavioral therapy |
| Wagena EJ | 2005 | Netherlands | COPD | 23 | NA | 300 | 86 | placebo | 89 | counseling |
| Zellweger J | 2005 | Europe | healthy | > 10 | 26 | 300 | 517 | Placebo | 170 | counseling |
| Evins AE | 2005 | USA | schizophrenia | ≧10 | NA | 300 | 25 | placebo | 28 | cognitive-behavioral therapy |
| Holt S | 2005 | New Zealand | healthy | ≧10 | Past 1 yr | 300 | 88 | Placebo | 46 | counseling |
| Myles PS | 2004 | Australia | on surgery waiting list | ≧10 | NA | 300 | 24 | placebo | 23 | educational program |
| Simon JA | 2004 | USA | healthy | ≧20 | 39 | 300 | 121 | Placebo | 123 | counseling + nicotine patch |
| Aubin HJ | 2004 | France | healthy | ≧10 | Past 1 yr | 300 | 340 | placebo | 164 | brief counseling |
| Killen JD | 2004 | England | adolescent | ≧10 | NA | 150 | 103 | placebo | 108 | Nicotine patch |
| Dalsgareth OJ | 2004 | Denmark | healthy | ≧10 | 26 | 300 | 222 | placebo | 114 | no |
| Hatsukami DK | 2004 | USA | healthy | ≧20 | >3mon in pre 1 year | 300 | 295 | placebo | 299 | counseling |
| Swanson NA | 2003 | USA | healthy | ≧5 | 10 | NA | 30 | counseling | 50 | no |
|  |  |  |  |  |  | NA | 30 | No Tx. | 30 | nicotine patch |
| Tonnesen P | 2003 | Europe | healthy | ≧10 | 30 | 300 | 527 | Placebo | 180 | counseling |
| Tonstad S | 2003 | Europe | CVD | ≧10 | 49 | 300 | 315 | Placebo | 314 | Brief motivational support |
| Hurt RD | 2003 | USA | healthy | ≧15 | Past 1 yr | 300 | 96 | placebo | 98 | Brief message to stop smoking |
|  |  |  |  |  |  | 300 | 88 | placebo | 88 |  |
| Lerman C | 2002 | USA | healthy | ≧10 | NA | 300 | 229 | placebo | 197 | counseling |
| Hall SM | 2002 | USA | healthy | ≧10 | 20 | 300 | 36 | Placebo | 37 | medical management |
|  |  |  |  |  |  |  | 37 |  | 36 | psychological intervention |
| Killen JD | 2006 | Euro. | healthy | ≧10 | NA | 150 | 181 | Placebo | 181 | counseling |
| Lerman C | 2002 | USA | healthy | ≧10 | NA | 300 | 128 | placebo | 123 | behavioral counseling |
| George TP | 2002 | USA | schizophrenia | 24 | NA | 300 | 16 | placebo | 16 | psycho education |
| Evins AE | 2001 | USA | schizophrenia | 30 | 40 | 300 | 9 | placebo | 9 | cognitive behavioral counseling |
| Hays JT | 2001 | USA | healthy | ≧15 | Past 1 yr | 300 | 214 | Placebo | 215 | counseling |
| Gonzales DH | 2001 | USA | healthy | ≧15 | past 1mon | 300 | 226 | placebo | 224 | Brief individual counseling |
| Tashkin D | 2001 | USA | COPD | ≧15 | 51 | 300 | 204 | placebo | 200 | counseling |
| Hertzberg MA | 2001 | USA | chronic posttraumatic stress disorder | NA | NA | 300 | 10 | placebo | 5 | Personalized message |
| Jorenby DE | 1999 | USA | healthy | ≧15 | ≧25 | 300 | 244 | Placebo | 160 | no |
|  |  |  |  |  |  | 300 | 245 | No Tx. | 244 | Nicotine patch |
| Hurt RD | 1997 | USA | healthy | ≧15 | Past 1 yr | 100 | 153 | Placebo | 153 | Brief individual counseling |
|  |  |  |  |  |  | 150 | 153 |  |  |  |
|  |  |  |  |  |  | 300 | 156 |  |  |  |

**Supplementary Table 2. Characteristics of bupropion trials**

| **Author** | **Year** | **Country** | **character** | **Characteristics of Patient use of Cigarettes/D** | **Pack years** | **varenicline Dosage (Mg/d)** | **No. of Intervention Group** | **Tr. in Control group** | **No. of Control Group** | **Co-treatment in both group** |
| --- | --- | --- | --- | --- | --- | --- | --- | --- | --- | --- |
| Gonzales D | 2006 | USA | healthy | >=10 | 32 | 2 | 352 | placebo | 344 | counseling |
| Jorenby DE | 2006 | USA | healthy | >=10 | 25 | 2 | 344 | placebo | 341 | counseling |
| Tonstad S | 2006 | Europe | healthy | >=10 | 28 | 2 | 603 | placebo | 607 | no |
| Oncken C | 2006 | USA | healthy | >=10 | 25 | 1 | 259 | placebo | 129 | counseling |
|  |  |  |  |  |  | 2 | 259 |  |  |  |
| Nides M | 2006 | USA | healthy | >=10 | 24 | 0.3 | 128 | placebo | 123 | counseling |
|  |  |  |  |  |  | 1 | 128 |  |  |  |
|  |  |  |  |  |  | 2 | 127 |  |  |  |
| Burstein, AH | 2006 | USA | healthy | >=10 | 50 | 1 | 8 | placebo | 8 | no |
|  |  |  |  |  |  | 2 | 8 |  |  |  |
| Williams KE | 2007 | USA | healthy | >=10 | 30 | 2 | 251 | placebo | 126 | counseling |
| Tsai, S | 2007 | Asia | healthy | >=10 | 20 | 2 | 126 | placebo | 124 | counseling |
| Nakamura M | 2007 | Japan | healthy | >=10 | 20 | 0.5 | 153 | placebo | 154 | counseling |
|  |  |  |  |  |  | 1 | 156 |  |  |  |
|  |  |  |  |  |  | 2 | 156 |  |  |  |
| Niaura R | 2008 | USA | Healthy | >=10 |  | 0.5-2 | 157 | Placebo | 155 | none |
| Aubin | 2008 | Europe/US | Healthy | >=10 |  | 1 | 376 | NRT | 370 | none |

**Supplementary Table 3. Characteristics of varenicline trials**
